# Synthesis and Hydrolytic Degradation of Substituted Poly(DL-Lactic Acid)s

**DOI:** 10.3390/ma4081384

**Published:** 2011-08-10

**Authors:** Hideto Tsuji, Takehiko Eto, Yuzuru Sakamoto

**Affiliations:** Department of Environmental and Life Sciences, Graduate School of Technology, Toyohashi University of Technology, Tempaku-cho, Toyohashi, Aichi 441-8580, Japan; E-Mails: takehicollege@hotmail.co.jp (T.E.); y043817@edu.imc.tut.ac.jp (Y.S.)

**Keywords:** substituted poly(lactide)s, poly(2-hydroxyalkanoate), polymerization hydrolytic degradation, thermal degradation

## Abstract

Non-substituted racemic poly(DL-lactic acid) (PLA) and substituted racemic poly(DL-lactic acid)s or poly(DL-2-hydroxyalkanoic acid)s with different side-chain lengths, *i.e*., poly(DL-2-hydroxybutanoic acid) (PBA), poly(DL-2-hydroxyhexanoic acid) (PHA), and poly(DL-2-hydroxydecanoic acid) (PDA) were synthesized by acid-catalyzed polycondensation of DL-lactic acid (LA), DL-2-hydroxybutanoic acid (BA), DL-2-hydroxyhexanoic acid (HA), and DL-2-hydroxydecanoic acid (DA), respectively. The hydrolytic degradation behavior was investigated in phosphate-buffered solution at 80 and 37 °C by gravimetry and gel permeation chromatography. It was found that the reactivity of monomers during polycondensation as monitored by the degree of polymerization (*DP*) decreased in the following order: LA > DA > BA > HA. The hydrolytic degradation rate traced by *DP* and weight loss at 80 °C decreased in the following order: PLA > PDA > PHA > PBA and that monitored by *DP* at 37 °C decreased in the following order: PLA > PDA > PBA > PHA. LA and PLA had the highest reactivity during polymerization and hydrolytic degradation rate, respectively, and were followed by DA and PDA. BA, HA, PBA, and PHA had the lowest reactivity during polymerization and hydrolytic degradation rate. The findings of the present study strongly suggest that inter-chain interactions play a major role in the reactivity of non-substituted and substituted LA monomers and degradation rate of the non-substituted and substituted PLA, along with steric hindrance of the side chains as can be expected.

## 1. Introduction

Poly(hydroxyalkanoate)s [*i.e*., poly(hydroxyalkanoic acid)s or PHAs] such as polylactide [*i.e*., poly(lactic acid)], poly(glycolide) [*i.e*., poly(glycolic acid)], poly(3-hydroxybutyrate) [*i.e*., poly(3-hydroxybutanoic acid)], poly(ε-caprolactone) [*i.e*., poly(6-hydroxyhexanoic acid)], and their copolymers have been used as scaffolds and pharmaceutical materials whose hydrolytic degradation behavior *in vivo* should be accurately manipulated [1,2,3,4,5,6,7]. Polylactide and polyglycolide are kinds of poly(2-hydroxyalkanoic acid); poly(3-hydroxybutyrate) is a kind of poly(3-hydroxyalkanoic acid); and poly(ε-caprolactone) is a kind of poly(6-hydroxyalkanoic acid). The degradation *in vivo* and enzymatic and non-enzymatic hydrolytic degradation *in vitro* of poly(lactic acid)—a PHA—have been intensively researched [7]; poly(lactic acid) has also been investigated in terms of its molecular weight [8,9,10,11,12], tacticity [8,11,12,13,14], comonomer type [13,15,16,17,18,19], additives such as polymers [20,21,22,23,24,25,26,27,28,29,30,31], crystallinity [32,33,34], crystalline size [35,36,37,38,39], orientation [40,41,42,43,44], and material morphology [45,46,47]. The molecular weight and comonomer type are crucial parameters for determining the hydrolytic degradation rate and mechanism of poly(lactic acid) [7]. The hydrolytic degradation rate has been found to increase by decreasing the molecular weight and to decrease by incorporating a hydrophilic comonomer.

**Figure 1:**
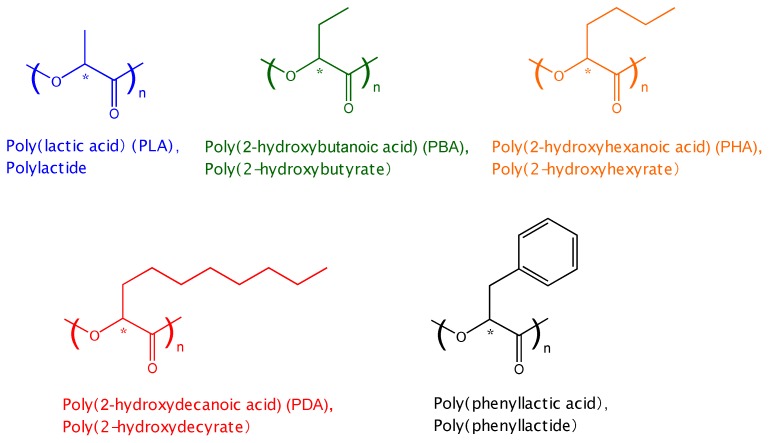
Molecular structures of substituted and non-substituted poly(DL-lactic acid)s (PLAs).

Poly(2-hydroxybutyrate) also known as poly(2-hydroxybutyric acid) or poly(2-hydroxybutanoic acid [P(2HB)] can be synthesized from 2-hydroxybutanoic acid with a chiral α-carbon. The structure of P(2HB) (Figure 1) is similar to that of PLA, the methyl group of the latter being substituted with an ethyl group. Baker *et al.* intensively synthesized a wide variety of substituted PLAs, including P(2HB), and investigated their physical properties and thermal degradation [48,49,50]. In their study, most of the synthesized polymers were racemic and noncrystallizable. Tsuji *et al.* synthesized optically active phenyl-substituted PLA (Figure 1) and its copolymers with lactic acid and investigated their crystallization behavior and thermal properties [51]. They also synthesized optically active and crystallizable poly(L-2-hydroxybutyrate) [P(L-2HB)] and poly(D-2-hydroxybutyrate) [P(D-2HB)] and found that stereocomplexation occurs in the blends of P(L-2HB) and P(D-2HB), which enhances the crystallization and hydrolytic/thermal degradation resistance compared to intact P(L-2HB) or P(D-2HB) [52,53]. Very recently, they found that hetero-stereocomplexation occurs between poly(D-lactic acid) (PDLA) and P(L-2HB) or between poly(L-lactic acid) (PLLA) and P(D-2HB) [54]. Interestingly, PDLA is reported to have a stronger interaction with the L-form of phenyl-substituted PLA than with the D-form [55].

However, in spite of numerous studies on the hydrolytic degradation behavior of PLA and PGA and their copolymers [7], few reports can be found for other P(2HB)s. Reported examples for P(2HB) are those of enzymatic and nonenzymatic degradation of poly(lactic acid-co-mandelic acid or phenyllactic acid), which are copolymers with lactic acid units, methyl groups of which are substituted with aromatic groups [56,57,58]. To the best of our knowledge, no report on hydrolytic degradation of PLA homopolymers, methyl groups of which are substituted with other aliphatic groups, has been published so far. In the present study, we synthesized PLA homopolymers, the methyl groups of which are substituted with other aliphatic groups, such as ethyl, butyl, or octyl groups, as well as non-substituted PLA homopolymer (Figure 1) by a conventional polycondensation method and investigated the hydrolytic degradation behavior of the substituted and non-substituted PLA homopolymers. Here, we used racemic DL-monomers to obtain noncrystallizable substituted and non-substituted PLA homopolymers to exclude the effects of highly ordered structures formed by crystallization during specimen preparation and hydrolytic degradation on hydrolytic degradation behavior.

## 2. Experimental Section

### 2.1. Materials

Substituted and non-substituted PLA polymers were synthesized by polycondensation or step-growth polymerization of DL-lactic acid (abbreviated as LA, Wako Pure Chemical Industries, Ltd., Tokyo, Japan), DL-2-hydroxybutanoic acid (butyric acid or BA) (Tokyo Chemical Industry Co., Ltd., Tokyo, Japan), DL-2-hydroxyhexanoic acid or HA (Sigma-Aldrich Co., St. Louis, MO), and DL-2-hydroxydecanoic acid or DA (Wako Pure Chemical Industries, Ltd., Tokyo, Japan) at 130 °C with 2.5 wt% of *p*-toluenesulfonic acid monohydrate (Nacalai Tesque, Inc., Kyoto, Japan) under a constant nitrogen gas flow. For monitoring synthesis, the polymerization of monomers was performed at atmospheric pressure for 5 h (first polymerization step) and then under reduced pressure of 2.7 kPa (20 mmHg) for up to 24 h (second polymerization step) [51,52]. For the synthesis of polymers having number-average molecular weight (*M*_n_) of several thousands for hydrolytic degradation experiments, the second polymerization step was carried out for 24 h. The synthesized polymers from BA, HA, and DA (PBA, PHA, and PDA, respectively) were purified with precipitation with chloroform and methanol/water (v/v = 1/1) as a solvent and non-solvent. The synthesized polymer from LA (PLA) was purified by the same procedure, although acetone was used as the solvent instead of chloroform. The precipitated polymers were dried under reduced pressure for at least 7 days. The purified PLA was a fragile solid, whereas PBA, PHA, and PDA were highly viscous liquids.

### 2.2. Hydrolytic Degradation

Hydrolytic degradation of each sample (20 mg) was performed using the purified polymers in 100 mL of phosphate-buffered solution (pH 7.4) at 80 °C or 37 °C. After hydrolytic degradation, the samples were rinsed with fresh distilled water, soaked in fresh distilled water for 1 h, soaked for another 3 h in a new batch of fresh distilled water, and then dried under reduced pressure for at least 7 days. Although the polymerization and hydrolytic degradation were performed once in the present study, the subsequent investigations indicated similar dependences of the rates of polymerization [at fixed catalyst concentrations (mol%) and under atmospheric pressure] and hydrolytic degradation on the monomer type.

### 2.3. Measurements

The respective weight and number-average molecular weights (*M*_w_ and *M*_n_, respectively) of polymers were evaluated in chloroform at 40 °C by a Tosoh (Tokyo, Japan) GPC system with two TSK gel columns (GMH_XL_) using polystyrene standards. Therefore, the molecular weights are relative to polystyrene.

## 3. Results

### 3.1. Synthesis

**Figure 2:**
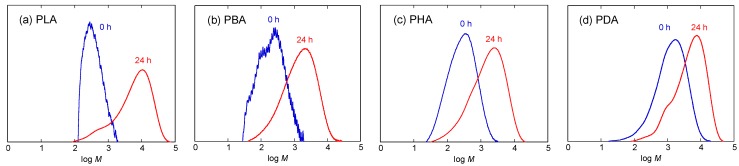
Molecular weight distribution curves of PLA (**a**); poly(DL-2-hydroxybutanoic acid) (PBA) (**b**); poly(DL-2-hydroxyhexanoic acid) (PHA) (**c**); and poly(DL-2-hydroxydecanoic acid) (PDA) (**d**) before the second polymerization step (0 h) and after polymerization under reduced pressure for 24 h.

**Figure 3:**
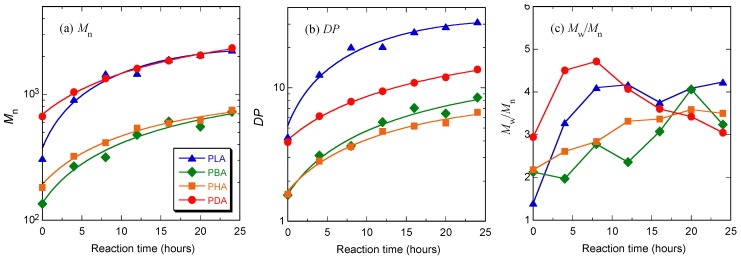
*M*_n_ (**a**), *DP* estimated from *M*_n_ (**b**), and molecular weight distribution (*M*_w_/*M*_n_) (**c**) of PLA, PBA, PHA, and PDA during the second polymerization step under reduced pressure at different times, as a function of reaction time.

In the first polymerization step, each monomer was polymerized by polycondensation to yield its oligomers at 130 °C at atmospheric pressure for 5 h to have a sufficiently high molecular weight not to be removed under reduced pressure in the second polymerization step. In the second polymerization step, thus formed oligomers were further polymerized by polycondensation under reduced pressure at 130 °C for 24 h [50,51]. Figure 2 shows the changes in molecular weight distribution curves of PLA, PBA, PHA, and PDA during the second polymerization under reduced pressure. As seen, whole curves shifted to a higher molecular weight with the second polymerization. Table 1 and Figure 3 show *M*_n_, molecular weight distribution (*M*_w_/*M*_n_), the degree of polymerization estimated from *M*_n_, (*DP*), and *DP*_a_ − *DP*_b_ of PLA, PBA, PHA, and PDA during the first polymerization step of the monomers at atmospheric pressure and the second polymerization step under reduced pressure. Here, *DP*_a_ and *DP*_b_ are *DP* before and after polymerization, respectively, and *DP*_b_ for the first polymerization step is one. As seen in Table 1 and Figure 3, the *DP* and *DP*_a_ − *DP*_b_ of polymers after the first polymerization step for 5 h and the second polymerization step for 24 h decreased in the following orders: PLA > PDA > PHA ≅ PBA and PLA > PDA > PBA > PHA, respectively. The catalytic *p*-toluenesulfonic acid concentrations for LA, BA, HA, and DA during polymerization per mol monomer were 1.18, 1.37, 1.74, and 2.61 × 10^−2^ (mol/mol). The catalytic concentration and, therefore, its effect should decrease in the following order: DA > HA > BA > LA. However, the highest *DP*_a_ − *DP*_b_ value of PLA despite the lowest catalytic concentration for LA and the lowest *DP*_a_ − *DP*_b_ value of PHA despite the second highest catalytic value for HA should be due to the highest and lowest reactivities of LA and HA, respectively. For reference, we calculated the *DP*_a_ − *DP*_b_ relative to the catalytic concentrations (mol/mol). The calculated values were 277, 42, 34, and 112 (mol/mol) for PLA, PBA, PHA, and PDA after the first polymerization step for 5 h and 2273, 503, 285, and 376 (mol/mol) after the second polymerization step for 24 h. These values and, therefore, the actual reactivity values of the monomers in the presence of *p*-toluenesulfonic acid decreased in the following order: LA > DA > BA > HA for the first step and LA > BA > DA > HA for the second step. The former trend is consistent with the aforementioned non-normalized *DP* and *DP*_a_ − *DP*_b_ values after the second polymerization step.

**Table 1:** Number-average molecular weight (*M*_n_), molecular weight distribution (*M*_w_/*M*_n_), and degree of polymerization (*DP*) of 2-hydroxyalkanoic acid homopolymers after the first polymerization step at atmospheric pressure for 5 h and subsequent second polymerization for 24 h under reduced pressure.

Code	After first polymerization step at atmospheric pressure for 5 hours					After second polymerization step under reduced pressure for 24 hours			
	*M*_n_^a)^	*M*_w_/*M*_n_^a)^	*DP*	*DP*_a_ − *DP*_b_^b)^		*M*_n_^a)^	*M*_w_/*M*_n_^a)^	*DP*	*DP*_a_ − *DP*_b_^b)^
PLA	309	1.40	4.29	3.29 (4.29–1)		2250	4.23	31.2	26.9 (31.2–4.3)
PBA	135	2.13	1.56	0.56 (1.56–1)		728	3.24	8.46	6.90 (8.46–1.56)
PHA	182	2.18	1.59	0.59 (1.59–1)		748	3.50	6.55	4.96 (6.55–1.59)
PDA	668	2.95	3.92	2.92 (2.92–1)		2340	3.05	13.7	9.8 (13.7–3.9)

^a)^ *M*_n_ and *M*_w_ are the number- and weight-average molecular weights, respectively.

^b)^ *DP*_b_ and *DP*_a_ are *DP* before and after polymerization, respectively. The actual calculation is shown in the parentheses.

### 3.2. Hydrolytic Degradation at 80 °C

**Figure 4:**
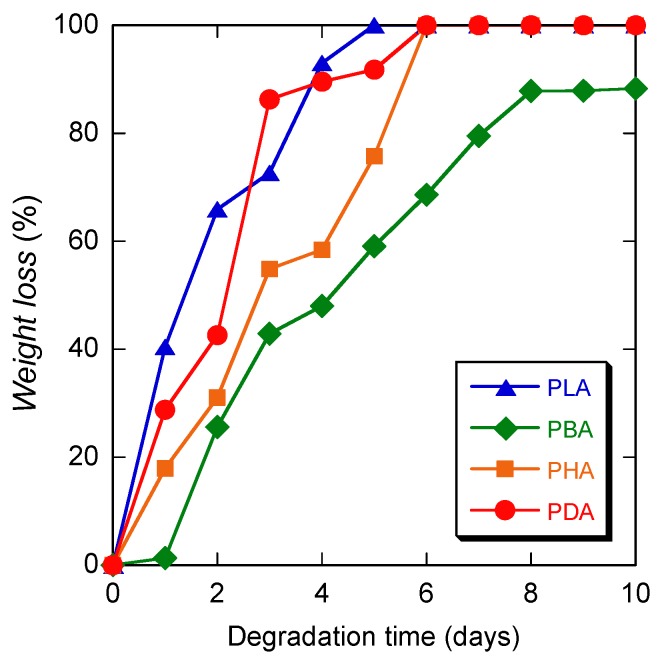
Weight loss of PLA, PBA, PHA, and PDA during hydrolytic degradation at 80 °C, as a function of degradation time.

Figure 4 shows the weight loss values of PLA, PBA, PHA, and PDA during hydrolytic degradation at 80 °C as a function of degradation time. The weight loss is an indicator of the fraction of water-soluble oligomers and monomers that are formed by hydrolytic degradation and diffused out of the mother material. The weight loss of all polymers increased monotonically with degradation time, without any induction period. The rate of weight loss, with the exception of PDA at 3 days, decreased in the following order: PLA > PDA > PHA > PBA. This trend is consistent with that for the reactivity of the monomers, with the exception of PHA. The higher values of PHA compared to those of PBA may be ascribed to the lowest starting *DP* value of 8.7 (Table 2).

**Table 2:** *M*_n_, *M*_w_/*M*_n_, and *DP* of PLA, PBA, PHA, and PDA after hydrolytic degradation at 80 °C for 4 days and at 37 °C for 28 days.

Code	After Purification (Before hydrolytic degradation)				After hydrolytic degradation at 80 °C for 4 days				After hydrolytic degradation at 37 °C for 28 days		
	*M*_n_^a)^	*M*_w_/*M*_n_^a)^	*DP*		*M*_n_^a)^	*M*_w_/*M*_n_^a)^	*DP*		*M*_n_^a)^	*M*_w_/*M*_n_^a)^	*DP*
PLA	3850	2.46	53.5		350	1.92	4.85		856	6.02	11.9
PBA	861	3.05	10.0		450	2.28	5.23		573	3.22	6.66
PHA	991	2.77	8.68		386	2.17	3.38		657	3.30	5.75
PDA	2910	3.66	17.1		799	2.39	4.69		1436	3.54	8.43

^a)^ *M*_n_ and *M*_w_ are the number- and weight-average molecular weights, respectively.

**Figure 5:**
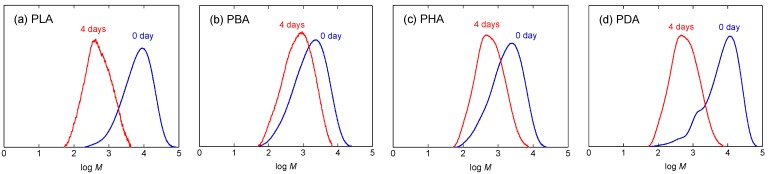
Molecular weight distribution curves of PLA (**a**), PBA (**b**), PHA (**c**), and PDA (**d**) before hydrolytic degradation (0 days) and after hydrolytic degradation at 80 °C for 4 days.

**Figure 6:**
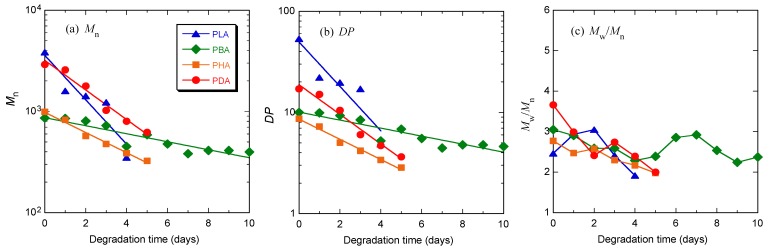
*M*_n_ (**a**), *DP* estimated from *M*_n_ (**b**), and molecular weight distribution (*M*_w_/*M*_n_) (**c**) of PLA, PBA, PHA, and PDA, during hydrolytic degradation at 80 °C, as a function of degradation time.

Figure 5 shows the molecular weight distribution curves of PLA, PBA, PHA, and PDA before hydrolytic degradation and after hydrolytic degradation at 80 °C for 4 days. Table 2 and Figure 6 show *M*_n_, *DP* estimated from *M*_n_, and molecular weight distribution (*M*_w_/*M*_n_) of PLA, PBA, PHA, and PDA during hydrolytic degradation. The degradation rate was calculated according to the following equation [59]:

(1) $$\ln DP(t_{2})=\ln DP(t_{1})-k(t_{2}-t_{1}),$$

where *DP*(*t*_2_) and *DP*(*t*_1_) are *DP* values at degradation times of *t*_2_ and *t*_1_, respectively. The *k* values thus obtained 0.506, 0.091, 0.230, and 0.337 day^−1^ for PLA, PBA, PHA, and PDA, respectively. The *k* values of the polymers decreased in the following order: PLA > PDA > PHA > PBA. Owing to the difficulty of synthesis of substituted PLA homopolymers including poly(mandelic acid), the hydrolytic or enzymatic degradation rates or *k* values have been reported only for the copolymers of LA with BA, DL-2-hydroxy-3-methylbutanoic acid, DL-2-hydroxy-4-methylpentanoic acid, phenyllactic acid, or mandelic acid [56,57,58]. Most *M*_w_/*M*_n_ values were in the range of 2 to 3. This is in marked contrast with crystalline PLLA, which showed *M*_w_/*M*_n_ values over 4 [34].

### 3.3. Hydrolytic degradation at 37 °C

**Figure 7:**
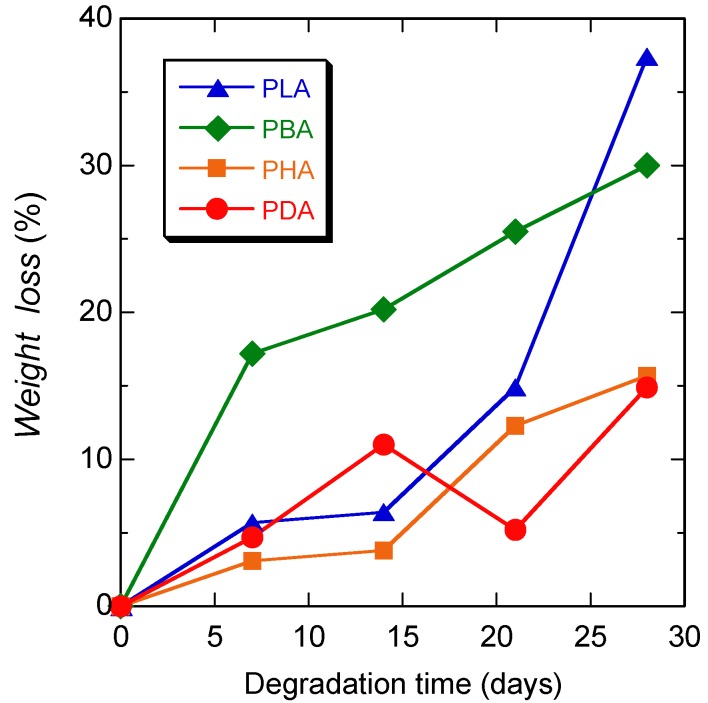
Weight loss of PLA, PBA, PHA, and PDA during hydrolytic degradation at 37 °C, as a function of degradation time.

Figure 7 shows the weight loss values of PLA, PBA, PHA, and PDA during hydrolytic degradation at 37 °C as a function of degradation time. Similar to the weight loss at 80 °C, the weight loss of all polymers at 37 °C increased monotonically with degradation time, without any induction period. For the degradation period of 7–21 days, PBA showed the highest weight loss, whereas PLA, PHA, and PDA showed similar weight losses that were lower. The weight loss of PLA was highest at a degradation period of 28 days. Due to the low and dispersed weight loss values at 37 °C, the hydrolytic degradation rate at that temperature is not discussed here.

**Figure 8:**
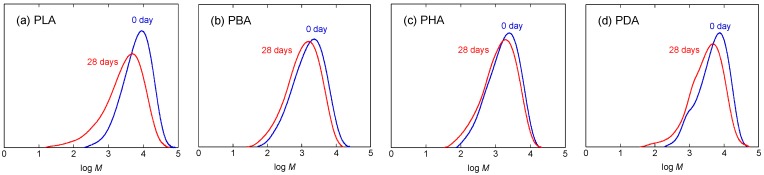
Molecular weight distribution curves of PLA (**a**), PBA (**b**), PHA (**c**), and PDA (**d**) before hydrolytic degradation (0 days) and after hydrolytic degradation at 37 °C for 28 days.

**Figure 9:**
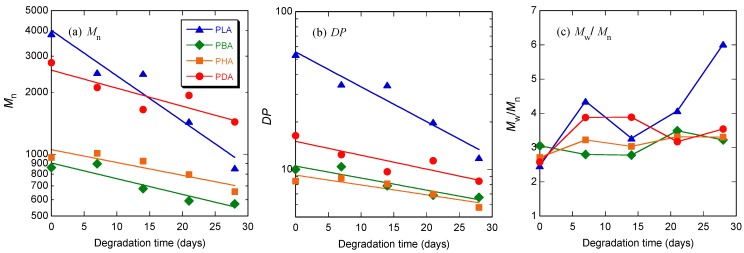
*M*_n_ (**a**), *DP* estimated from *M*_n_ (**b**), and molecular weight distribution (*M*_w_/*M*_n_) (**c**) of PLA, PBA, PHA, and PDA during hydrolytic degradation at 37 °C, as a function of degradation time.

Figure 8 shows the molecular weight distribution curves of PLA, PBA, PHA, and PDA before hydrolytic degradation (0 day) and after hydrolytic degradation at 37 °C for 28 days. Figure 9(a)–(c) shows *M*_n_, *DP* estimated from *M*_n_, and *M*_w_/*M*_n_ of PLA, PBA, PHA, and PDA during hydrolytic degradation. The *k* values calculated according to equation (1) were 5.08, 1.76, 1.44, 2.02 × 10^−2^ day^−1^, for PLA, PBA, PHA, and PDA, respectively. The *k* values decreased in the following order: PLA > PDA > PBA > PHA. This trend is consistent with the reactivity of the monomers.

## 4. Discussion

The hydrolytic degradation rate is known to vary with molecular and highly ordered structures and material shapes [1,2,3,4,5,6,7,60]. In the present study, amorphous poly(DL-2-hydroxyalkanoic acid)s were used to eliminate the effects of highly ordered structures such as crystallinity. The molecular weight, the structures of terminal groups, and material shape are crucial for the hydrolytic degradation [60]. All the poly(DL-2-hydroxyalkanoic acid)s were synthesized by the same method and, therefore, the structures of terminal groups should be identical. For precise comparisons of the hydrolytic degradation rates of poly(DL-2-hydroxyalkanoic acid)s with different side chain lengths, the polymers with the similar *DP* values should be used. However, due to the differences in the reactivities of DL-2-hydroxyalkanoic acids with different side chain lengths, the poly(DL-2-hydroxyalkanoic acid)s had different *DP* values. Nevertheless, since polymerization and hydrolytic degradation are inverse reactions, the poly(DL-2-hydroxyalkanoic acid) synthesized from higher reactive monomers will have higher hydrolytic degradation rates. In other words, poly(DL-2-hydroxyalkanoic acid) with higher *DP* values after polymerization or before hydrolytic degradation will have higher hydrolytic degradation rates. The hydrolytic degradation rate decreased in the following order: PLA > PDA > PBA > PHA, which is consistent with that of monomer reactivity traced by *DP*: LA > DA > BA > HA (or HA > BA). This result suggests that the effects of monomer unit structure dominate those of *DP*.

If the rates of polymerization and hydrolytic degradation solely depend on the steric hindrance of side groups of the monomers and polymers, *i.e*., methyl, ethyl, butyl, and octyl groups for LA and PLA, BA and PBA, HA and PHA, and DA and PDA, respectively, the reaction rate should decrease in the following order: PLA (LA) > PBA (BA) > PHA (HA) > PDA (DA). This trend is based on the assumption that steric hindrance disturbs the formation and hydrolytic degradation of ester groups. However, the relative reaction rate when monitored by *DP* or weight loss in the present study was found to be PLA (LA) > PDA (DA) > PBA (BA) > PHA (HA) [or PHA (HA) > PBA (BA)]. This indicates that there must be an additional factor enhancing the polymerization and hydrolytic degradation of DA and PDA. It is probable that the weakest inter-chain interaction of PDA due to its largest side chains may have assisted in the removal of water molecules from the formed mixtures of oligomers and the diffusion of water molecules to the ester groups during hydrolytic degradation, although the highest hydrophobicity of PDA with the longest octyl side groups may help in the removal of water molecules during polymerization but disturbs the approach of water molecules during hydrolytic degradation. As stated in a review article [60], hydrophilicity and inter-chain interactions, which can be monitored by water absorption and glass transition temperature, will affect the rates of polymerization and hydrolytic degradation. However, these two indicators could not be measured in the present study due to the low molecular weights of the synthesized polymers. Further detailed studies using the polymers with high and similar molecular weights are required to investigate exact causes for the different reactivities of polymers.

## 5. Conclusions

The reactivity of monomers during polycondensation monitored by *DP* decreased in the following order: LA > DA > BA > HA, whereas the hydrolytic degradation rate traced by *DP* and weight loss at 80 °C decreased in the following order: PLA > PDA > PHA > PBA and that monitored by *DP* at 37 °C decreased in the following order: PLA > PDA > PBA > PHA. LA and PLA had the highest reactivity during polymerization and degradation rate, respectively, followed by DA and PDA; BA, HA, PBA, and PHA had the lowest reactivity and degradation rates. The findings of the present study strongly suggest that not only the steric hindrance of the side chains but also the inter-chain interaction determine the reactivity of the non-substituted and substituted LA monomers and the hydrolytic degradation rate of the non-substituted and substituted PLA.
